# Comparative Study of Phenolic Profiles, Antioxidant and Antiproliferative Activities in Different Vegetative Parts of Ramie (*Boehmeria nivea* L.)

**DOI:** 10.3390/molecules24081551

**Published:** 2019-04-19

**Authors:** Hong Wang, Caisheng Qiu, Ling Chen, Arshad Mehmood Abbasi, Xinbo Guo, Rui Hai Liu

**Affiliations:** 1 School of Food Science and Engineering, South China University of Technology, Guangzhou 510640, China; whongaq@163.com (H.W.); felchen@scut.edu.cn (L.C.); 2 Department of Food Science, Stocking Hall, Cornell University, Ithaca, NY 14853, USA; 3 Institute of Bast Fiber Crops, Chinese Academy of Agricultural Sciences, Changsha 410205, China; qcaisheng@163.com; 4 Department of Environment Sciences, COMSATS University Islamabad, Abbottabad 22060, Pakistan; arshad799@yahoo.com

**Keywords:** *Boehmeria nivea* L., phenolics, flavonoids, cellular antioxidant activities, antiproliferative properties

## Abstract

Ramie (*Boehmeria nivea* L.) is usually cultivated as a fiber crop, but it is also well known for its potential use in animal feeding with viable commercial applications. In this study, the phenolics profile as well as cellular antioxidant and antiproliferative activities were investigated in free and bound fractions of six different vegetative parts from *Boehmeria nivea* L. The highest total phenolic content was observed in bud (4585 ± 320 mg GAE/100 g DW), whereas root and petiole had the lowest total phenolic contents, 442.8 ± 9.8 and 630.9 ± 27.0 mg GAE/100 g DW, respectively. Likewise, phloem had the most abundant total flavonoids (2755 ± 184 mg CE/100 g DW), whereas the lowest flavonoid contents was found in root and petiole, 636.9 ± 44.2 and 797.4 ± 87.6 mg CE/100 g DW, respectively. Xylem and bud depicted remarkable antioxidant and antiproliferative activities, which could be explained by their diverse phenolic composition, especially chlorogenic acid and epicatechin. The *Boehmeria nivea* L. plant might be a valuable resource for high value-added phenolic compounds used in food and non-food industries.

## 1. Introduction

Ramie (*Boehmeria nivea* L.), known as “China grass”, is a perennial plant belonging to the Urticaceae family of *Boehmeria* genus. It is native to eastern Asia, including China, Japan and Malay Peninsula. China led in the production of ramie with 99,981 tons in 2016, which was about 97.6% of the world production [1]. Ramie is well known for its great potential use in animal feeding, but also provides natural fibers of excellent strength, elasticity, moisture absorption, and antibacterial properties in the textile industry [2,3].

The role of plant phenolics in the prevention against chronic diseases such as cardiovascular, diabetes and neurodegenerative diseases has been suggested in many studies [4,5,6]. Recent studies explored diversified health benefits of ramie leaves, such as antioxidant, anticancer, anti-inflammatory, anti-obesity, antibacterial activities, and hypoglycemic and hypolipidemic effects [7,8,9,10,11,12], which might be attributed to the bioactive phenolic compounds. Except for leaves, roots of ramie are also listed in the Chinese Pharmacopoeia, due to medicinal function in heat-clearing and detoxicating, hemostasis, and preventing miscarriage [13]. The bioactive phenolic compounds in ramie make it a potential candidate source of nutraceuticals and functional food. Besides, the phenolics in ramie might contribute to the good qualities of this plant in industrial applications such as being resistant against biodegradation, anti-bacterial properties, and protective against mold activity [9]. 

However, limited information about the phenolic compounds, antioxidant and antiproliferative activities of the different vegetative parts from *Boehmeria nivea* L. is available so far, and to our knowledge, there is none on xylem, phloem or petiole. Taking into consideration the potential of *Boehmeria nivea* L. for diverse uses, phenolic profiles, antioxidant and antiproliferative activities of the vegetative parts (root, xylem, phloem, petiole, leaves, and bud) were investigated in this study. These results will offer a scientific basis for the development of *Boehmeria nivea* L. as a valuable plant for food and industrial application.

## 2. Results and Discussion

### 2.1. Comparison of Phenolic Content in Different Parts of Boehmeria nivea L.

**Figure 1:**
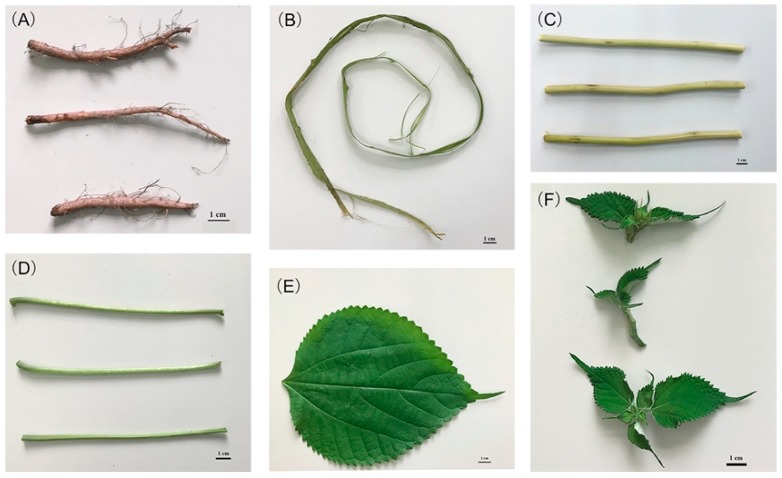
The photos of different vegetative parts including (**A**) root, (**B**) xylem, (**C**) phloem, (**D**) petiole, (**E**) leaf, and (**F**) bud from *Boehmeria nivea* L.

Six vegetative parts of *Boehmeria nivea* L. were collected including root, xylem, phloem, petiole, leaves, and bud, as shown in Figure 1. The highest moisture content was found in petiole (92.0%, *p* < 0.05), followed by bud (84.3%) and root (80.6%), while the other parts had lower moisture content (78.7 to 79.1 %) without significant differences. 

**Figure 2:**
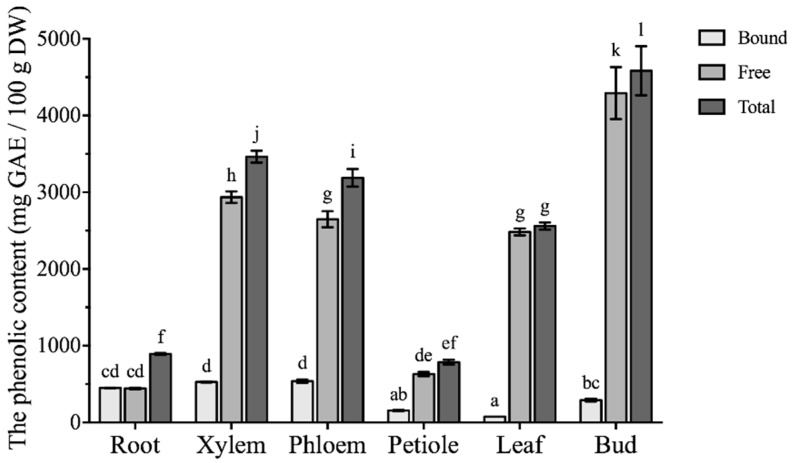
The phenolic contents of different vegetative parts from *Boehmeria nivea* L. (mean ± SD, n = 3). Bars with different letters indicate significant differences among the samples (*p* < 0.05).

Free and bound phenolic contents in different vegetative parts of *Boehmeria nivea* L. were expressed as milligrams of gallic acid equivalents per 100 g of dry weight (GAE/100 g DW), as presented in Figure 2. Bound phenolics in plant-derived foods are interesting sources of phenolics which are bound to the plant cell wall, and thus can be released in alkaline hydrolyses. In contrast, free phenolics were extractable by organic solvents in the mild condition. Thus, the total phenolics was the sum of bound and free phenolics. A notable variation was observed in bound, free and total phenolic contents among different vegetative parts of *Boehmeria nivea* L. Higher bound phenolic contents were found in phloem, xylem and root, with the amount ranging from 450.3 ± 3.8 to 539.7 ± 22.3 mg GAE/100 g DW. However, in leaves, petiole and bud, bound phenolics were between 76.30 ± 0.16 and 292.5 ± 19.3 mg GAE/100 g DW. The highest free phenolic content was found in the bud of *Boehmeria nivea* L. (4292 ± 339 mg GAE/100 g DW), whereas root and petiole had the lowest free phenolic content, 442.8 ± 9.8 and 630.9 ± 27.0 mg GAE/100 g DW, respectively. The contribution of free phenolics to the total phenolics in most vegetative parts was more than 80%, except root with the amount of 50%. Likewise, the highest total phenolic content was also observed in bud (4585 ± 320 mg GAE/100 g DW), which was over five times higher than the lowest measured levels in root and petiole. According to Chen et al., total phenolic content in the leaves of *Boehmeria nivea* L. on fresh weight basis was 413.3 mg GAE/100 g FW, which is comparable to our results (535.6 mg GAE/100 g FW) [11]. However, Wang et al. compared leaf extracts from 10 ramie cultivars ranging 0.52 to 2.41 mg GAE/g DW [7], which is relatively lower than our results. Such variations might be attributed to different extraction methods as well as climate, weather, water access, sunlight, and other physicochemical and geographical factors.

Phenolics are secondary metabolites which are not only involved in the reproduction and physiology of plant, but also associated with reduced risk of major chronic diseases due to their antioxidant, anticancer and anti-microbial activities [14,15,16]. A present finding indicated that abundant phenolics in bud, xylem and phloem could contribute to further utilization of *Boehmeria nivea* L. in cosmetics, textiles, and fiber reinforcements. Bound phenolics in plants are interesting sources of phytochemicals, which are bound to the plant cell wall and can be released by different types of hydrolyses techniques like alkaline and acid hydrolysis, enzymatic hydrolysis, microwave-assisted hydrolysis, ultrasound-assisted hydrolysis, and other assisted hydrolysis [17,18,19]. During the past decades, since the bound phenolics released by the gut microbiota may play important roles, the importance and bioactivities of bound phenolics have been recognized by more and more research [17]. In this study, higher contents of bound phenolics in xylem, phloem and root declared these parts as potent nutraceutical resources.

### 2.2. Flavonoid Contents in Different Parts of Boehmeria nivea L.

**Figure 3:**
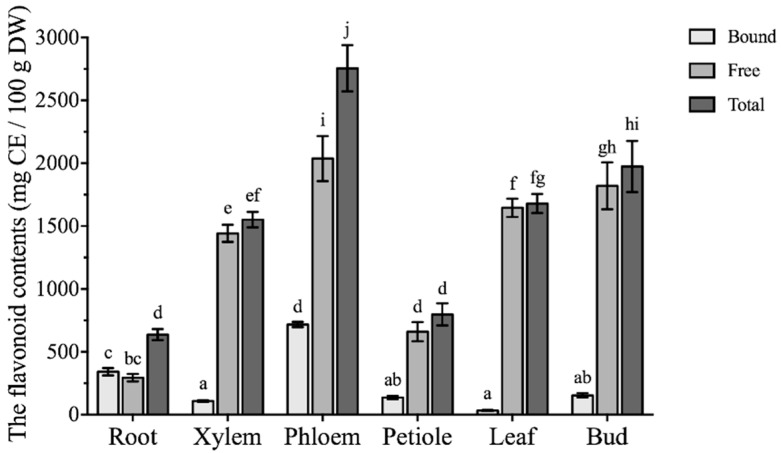
The flavonoid contents of different vegetative parts from *Boehmeria nivea* L. (mean ± SD, n = 3). Bars with different letters indicate significant differences among the samples (*p* < 0.05).

The contents of free and bound flavonoids are expressed as milligrams of catechin equivalent per 100 g of dry weight (CE/100g DW), showing notable variations among different parts of *Boehmeria nivea* L. (Figure 3). As for bound flavonoid contents, phloem and roots had the highest bound flavonoid contents (717.8 ± 20.5 and 342.1 ± 28.9 mg CE/100 g DW, respectively). Whereas, other parts of *Boehmeria nivea* L. had comparatively lower bound flavonoid contents which ranged from 34.23 ± 4.48 to 154.0 ± 17.2 mg CE/100 g DW. In the case of free flavonoids content, dramatic differences were noted among all six vegetative parts of *Boehmeria nivea* L. which ranged from 294.7 ± 30.3 to 2037 ± 179 mg CE/100 g DW. The highest content of free flavonoid contents was found in phloem, while the lowest one was calculated for root. Similar to the phenolics, the free flavonoids content was significantly higher than the bound flavonoid, i.e., from 2.8-fold in phloem to 48-fold in leaves. Likewise, phloem also had the most abundant total flavonoids (2755 ± 184 mg CE/100 g DW), followed by bud (1974 ± 203 mg CE/100 g DW), whereas the lowest flavonoid content was found in root and petiole, 636.9 ± 44.2 and 797.4 ± 87.6 mg CE/100 g DW, respectively. Total flavonoid contents in *Boehmeria nivea* L. leaves reported previously was about 210.4 mg CE/100 g FW [11], which is analogous to the results on fresh weight (FW) basis in our study (347.1 mg CE/100 g FW). Highest flavonoid content in phloem might explain the anti-microbial property of phloem fibers, leading to the potential utilization in textile, food or feed industry.

### 2.3. Phenolic Composition of Different Parts of Boehmeria nivea L.

Except for leaves, there is very limited information on the phenolic composition in other parts of *Boehmeria nivea* L. that can be better used as by-products. Six phenolic acids and four flavonoids were identified and quantified in *Boehmeria nivea* L. and data revealed potent variations among different parts (Table 1). 

Among the six phenolic acids, *p*-coumaric acid was the dominant phenolic compound in xylem, root, leaf and petiole, ranging from 344.1 ± 3.7 to 4155 ± 52 μg/g DW. Most of the *p*-coumaric acid content (80.1 to 99.0%) was contributed by the bound phenolic fraction. Beside this, a rich concentration of chlorogenic acid was found in the free fraction of xylem (1799 ± 25 μg/g DW). In contrast to *p*-coumaric acid and ferulic acid, a large portion of chlorogenic acid was presented in the free fraction, ranging from 75.5 to 100% of total amount in different parts of *Boehmeria nivea* L. Gallic acid and benzoic acid were not detected in petiole or leaf, and only a low amount of gallic acid was detected in xylem, phloem and bud, which ranged from 1.92 to 5.41 μg/g DW. 

Among the identified flavonoids, epicatechin was leading with 2540 ± 61.0 and 1459 ± 79.0 μg/g DW in the free fractions of xylem and phloem, respectively. Rutin was also among the most prevalent flavonoids in *Boehmeria nivea* L., which ranged from 18.82 ± 0.64 μg/g DW in petiole to 257.4 ± 9.7 μg/g DW in leaf. Another commonly distributed flavonoid was isoquercetin, as it was detected in most parts of *Boehmeria nivea* L., except phloem. Buds and leaves had the highest amount of isoquercetin (163.2 ± 12.7 and 101.0 ± 4.2 μg/g DW, respectively). Compared to other flavonoids, only low amounts of hyperoside were detected in root (48.32 ± 2.75 μg/g DW) and phloem (30.09 ± 0.50 μg/g DW). 

The majority of the phenolic compounds identified in the present study have already been reported in tested plant material previously [7,11,20]. However, previous authors were mainly focused on the composition of phenolics in leaves of *Boehmeria nivea* L. In all parts of the *Boehmeria nivea* L. except leaves, chlorogenic acid was also the most abundant phenolic acid in the free fractions of xylem, phloem, and bud. Besides, *p*-coumaric acid was the main phenolic acid in the bound fraction of xylem, root and bud (Table 1). Not only known as a nutraceutical for the prevention of metabolic syndrome, the anti-microbial properties of chlorogenic acid and *p*-coumaric acid can make these vegetative parts of *Boehmeria nivea* L. as a potential source of bioactive compounds in industrial application as preservatives and food additive [21,22,23,24].

### 2.4. Comparison of Antioxidant Activities among Different Parts of Boehmeria nivea L.

**Figure 4:**
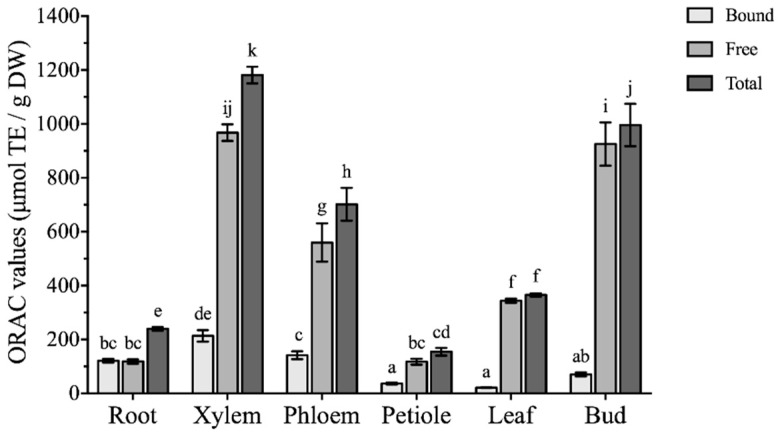
The ORAC values of different vegetative parts from *Boehmeria nivea* L. (mean ± SD, n = 3). Bars with different letters indicate significant differences among the samples (*p* < 0.05).

The antioxidant activity was first evaluated by ORAC assay (Figure 4), in different parts of *Boehmeria nivea* L. and measured levels were expressed as micromoles of trolox equivalent per gram of dry weight (TE/g DW). The total ORAC values showed notable variations (*p* < 0.05) among the different parts of *Boehmeria nivea* L., ranging from 239.9 ± 7.1 to 1181 ± 31 μmol TE/g DW. The highest total ORAC value was found in xylem, followed by bud, phloem, leaf, root, while petiole was the least. Comparatively, free fractions depicted high capacity to scavenge the oxygen radicals. The ORAC values for bound fractions ranged from 21.33 ± 1.62 to 213.5 ± 21.35 μmol TE/g DW, with maximum capacity in the xylem. In the free phenolic extracts, two parts of *Boehmeria nivea* L.: xylem and bud showed higher ORAC values at 967.8 ± 30.7 and 925.5 ± 80.3 μmol TE/g DW, respectively, followed by phloem (559.9 ± 71.2 μmol TE/g DW) and leaf (344.1 ± 7.5 μmol TE/g DW), while root and petiole had the lower ORAC values (118.4 ± 8.3 and 117.9 ± 10.9 μmol TE/g DW, respectively). The bound ORAC value in most vegetative parts accounted for less than 25% of the total ORAC values. However, the contribution of bound ORAC values to the total ORAC values in root could not be ignored, as it was comparable with ORAC values in the free fractions. 

**Figure 5:**
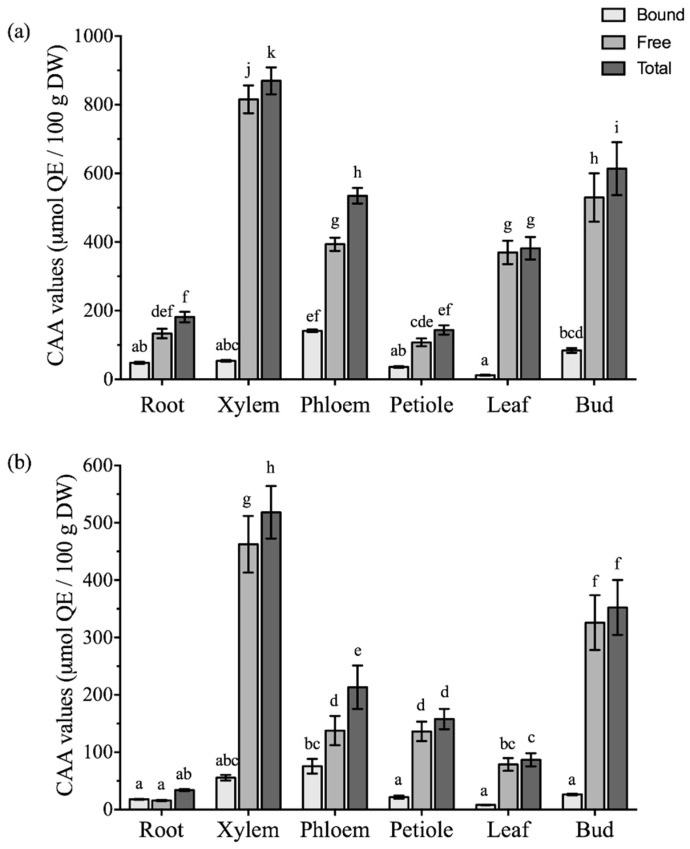
The CAA values of different vegetative parts from *Boehmeria nivea* L. in (**a**) no PBS wash protocol and (**b**) PBS wash protocol with bound and free fractions (mean ± SD, n = 3). Bars with different letters indicate significant differences among the samples (*p* < 0.05).

The antioxidant activity of different parts from *Boehmeria nivea* L. was further evaluated using cellular models to better simulate in vivo conditions [25]. The cellular antioxidant activity (CAA) was expressed as micromoles of quercetin per 100 g of dry weight (QE/100 g DW). In the no PBS wash protocol (Figure 5a), the bound fraction of phloem had the highest CAA value (141.2 ± 4.1 μmol QE/100 g DW). Other bound fractions from leaf (12.13 ± 1.19 μmol QE/100 g DW) to xylem (84.01 ± 6.62 μmol QE/100 g DW) were comparable. In the free extract of xylem, CAA values in the no PBS wash protocol were the highest (815.8 ± 40.5 μmol QE/100 g DW), followed by bud (529.7 ± 70.7 and 326.1 ± 47.8 μmol QE/100 g DW) and comparatively lower free CAA values were shown in the petiole and root (107.67 ± 11.63 and 133.5 ± 13.6 μmol QE/100 g DW, respectively). In the PBS wash protocol, a higher bound CAA values were also observed in phloem (75.61 ± 12.63 μmol QE/100 g DW) compared with other vegetative parts except xylem, ranging from 8.12 ± 0.39 μmol QE/100 g DW in leaf to 26.45 ± 1.40 μmol QE/100 g DW in bud. Xylem presented the highest free CAA values (462.71 ± 49.34 μmol QE/100 g DW), followed by bud (326.1 ± 47.8 μmol QE/100 g DW), as similar to the no PBS wash protocol, whereas root was the lowest one in the PBS wash protocol (16.06 ± 1.15 μmol QE/100 g DW). Due to the higher proportion of CAA in free fractions, the total CAA values presented a similar trend to the free ones. In no PBS and PBS wash protocols, the maximum levels of total CAA were found in xylem at 869.6 ± 39.2 μmol QE/100 g DW (no PBS wash) and 518.4 ± 45.8 μmol QE/100 g DW (PBS wash), followed by bud at 613.8 ± 77.1 (no PBS wash) and 352.5 ± 47.9 μmol QE/100 g DW (PBS wash), and phloem at 534.9 ± 22.8 (no PBS wash) and 213.5 ± 37.9 μmol QE/100 g DW (no PBS wash). In the no PBS wash protocol, petiole and root had lower total CAA values (143.7 ± 13.5 and 181.5 ± 15.3 μmol QE/100 g DW, respectively), and root was also the lowest one in the PBS wash protocol (34.06 ± 1.76 μmol QE/100 g DW).

**Figure 6:**
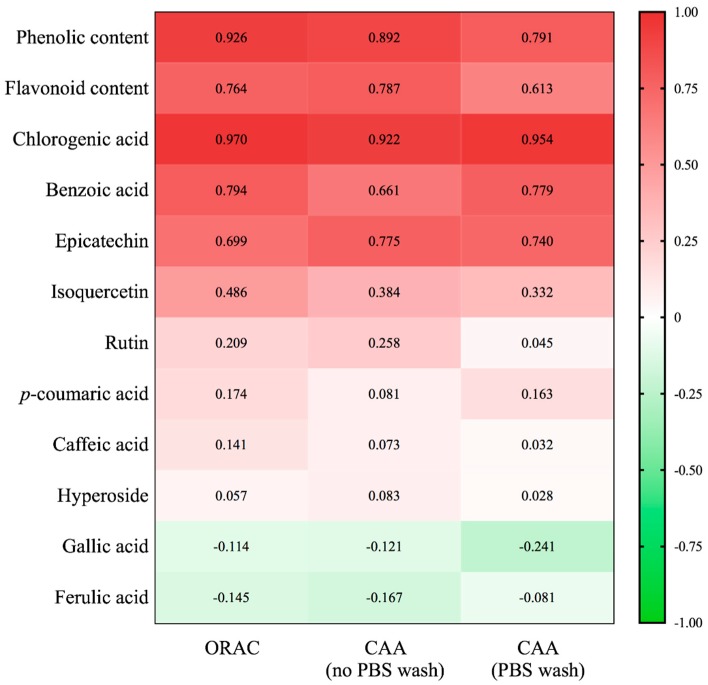
Heat map of Pearson`s correlation coefficient among phenolics and ORAC, CAA in no PBS wash and PBS wash protocols. Gradient color barcodes at the left indicated the minimum value in green and the maximum in red.

ORAC method is used to assess oxygen radical absorbance capacity [26], while the CAA assay reflects the absorption, uptake, metabolism, and distribution of antioxidants at the cellular level [25]. In general, the antioxidant capacity was positively correlated with the phenolic and flavonoid contents (Figure 6). The phenolic content exhibited strong positive association with ORAC (R^2^ = 0.926, *p* < 0.01) and CAA in both the no PBS wash (R^2^ = 0.892, *p* < 0.01) and PBS wash (R^2^ = 0.791, *p* < 0.01) protocols. However, less positive correlations were observed for the total flavonoids with ORAC (R^2^ = 0.764, *p* < 0.01) and CAA values in the no PBS wash (R^2^ = 0.787, *p* < 0.01) and PBS wash protocol (R^2^ = 0.613, *p* < 0.01). Among the phenolic compounds analyzed, the highest correlation with antioxidant activity was observed for chlorogenic acid (R^2^ = 0.922 to 0.970, *p* < 0.01) followed by benzoic acid and epicatechin (R^2^ = 0.661 to 0.794, *p* < 0.01). These results suggested that chlorogenic acid and epicatechin have good relations with the antioxidant activity of *Boehmeria nivea* L., which was consistent with previous reports [21,27].

### 2.5. Comparison of Antiproliferative Activities among Different Parts of Boehmeria nivea L.

Due to the higher contents of phenolics and antioxidant activities observed in free fractions of different parts from *Boehmeria nivea* L., the antiproliferative activities of free phenolic extract were further measured independently against human malignant melanoma cell A375, cervical carcinoma cell KB and hepatoma cell HepG2, and they were expressed as the median effective dose (EC_50_, mg DW/mL) (Table 2). 

Among the six parts, most of the free extracts effectively inhibited the cancer cell proliferation dose-dependently except petiole and leaf against A375 and KB cells. The phytochemical extracts of bud exhibited the strongest antiproliferative activities towards A375 cell with the lowest EC_50_ values (0.06 ± 0.01 mg DW/mL, *p* < 0.05), followed by xylem and root, while a weak antiproliferative activity towards A375 cells at higher doses was noted in phloem. The EC_50_ values of petiole and leaf could not be detected at the doses of soluble free extracts used in this experiment. As for KB cells, a similar trend was observed. Bud had the highest inhibitory effect with lowest EC_50_ (0.13 ± 0.01 mg DW/mL), followed by root, xylem, and finally phloem. The EC_50_ values against KB cells of petiole and leaf could not be determined at the doses of soluble free extracts used in this experiment. However, petiole and leaf exhibited inhibitory effects towards HepG2 cells, although they were weaker than the other four parts with similar antiproliferative effects (from 0.92 ± 0.14 to 1.41 ± 0.07 mg DW/mL). 

Similar to the comparison of antioxidant activities, the remarkable antiproliferative effects were found in bud and xylem (Table 2), which were consistent with its higher phenolic contents (Figure 2). Besides, root might be worth the resources of nutraceuticals and its active compounds, and therefore, deserves further investigation.

## 3. Materials and Methods 

### 3.1. Plant Materials 

*Boehmeria nivea* L. plant was collected from Guangzhou (China) at 113°17′ E longitude and 23°08′ N latitude in August of 2018. After cleaning, samples were separated into: root, xylem, phloem, petiole, leaf and bud, and stored at -80°C until analysis. The moisture content was determined by oven-drying method [28], by drying samples in an electric oven at 105 °C to the constant weight.

### 3.2. Preparation of Extracts

Free phenolic extract (FPE) of the different parts of *Boehmeria nivea* L. was made according to previous reports [11,29] with little modifications. Briefly, 20 g of root was blended with 200 mL chilled methanol for 5 min using a Waring Blender (DS-1, Shanghai Specimen and Model Factory, Shanghai, China). Then the mixture was homogenized on ice with a high-speed blender (Ultra-Turrax T25, IKA Works, Inc., Wilmington, NC) at 12000 rpm for 3 min. The homogenate was collected under reduced pressure through filter paper, and the residue was again extracted twice. The supernatants were pooled and evaporated at 45 °C. The free phenolic fractions were brought up to 10 mL with methanol. Other parts of *Boehmeria nivea* L. were extracted with a similar method to that mentioned above. Each sample was extracted in triplicate and extracts were stored at −40 °C until analysis.

Bound phenolic extract (BPE) was determinate as made earlier [30] with little modification. In brief, the residue from the free phenolic extraction was digested with 40 mL of 4 M sodium hydroxide at room temperature for 1 h with shaking. The mixture was acidified to pH 2 by using concentrated hydrochloric acid and then extracted with ethyl acetate at least six times until the supernatants turned colorless. The bound phenolic fractions obtained after the ethyl acetate were evaporated and were reconstituted up to 10 mL with methanol. The extractions were performed in triplicate, and extracts were maintained at −40 °C until the analysis.

### 3.3. Determination of Total Phenolic and Flavonoid Contents

Total phenolic content was determined using the Folin-Ciocalteu method as previously described [31,32]. The results were calculated according to the standard curve of gallic acid and expressed as mg gallic acid equivalents (GAE)/100 g sample (dry weight, DW). Total flavonoid content was analyzed using the previously described sodium borohydride/chloranil colorimetric method [33,34]. The results were calculated by comparing with the standard curve of fresh catechin and expressed as mg of catechin equivalents (CE)/100 g sample.

### 3.4. HPLC-PAD Analysis of Phenolic Compounds

The phenolic composition of the FPE and BPE were analysed using a Waters series HPLC system (Waters Co., Milford, MA, USA) equipped with a binary pump (model 1525), a micro degasser, an autosampler (model 2707), a thermostatically controlled column apartment (model 1500), and a photodiode array detector (model 2998) as described previously [32]. The samples were separated at 35 °C in a Waters XSelect^®^ HSS T3 column (5 μm, 4.6 × 150 mm) using a flow of 1 mL/min. The mobile phase consisted of a mixture of 0.1% trifluoroacetic acid solution (A) and acetonitrile (B) with the following gradient: from 0 to 5 min, 10% B; from 5 to 20 min, 10% to 20% B; from 20 to 25 min, 20% B; from 25 to 30 min, 20% to 35% B; from 30 to 40 min, 35% to 90% B; from 40 to 50 min, 90% to 10% B; and from 50 to 60 min, 10% B. The detector was set at 215, 280 and 325 nm for different phenolic compounds, respectively. Chromatographic peaks were identified and quantified by comparing the retention times in specific UV spectra with external standards.

### 3.5. Oxygen Radical Absorbance Capacity (ORAC) Assay

In vitro antioxidant activity was performed using ORAC assay [26,35]. Briefly, 20 μL of appropriate concentration of extract or Trolox standards (6.25–50 μM) diluted by 75 mM phosphate buffer (PBS, pH 7.4) was added to 200 μL of 0.96 μM fluorescein, then maintained for 20 min at 37 °C. After the addition of 20 μL of 119.4 mM 2,2′-azobis-amidinopropane (ABAP), the reaction was performed for 35 cycles every 4.5 min at excitation of 485 nm and emission of 535 nm using the FilterMax F5 Multi-Mode Microplate Reader (Molecular Devices, San Jose, CA, USA). The results were calculated according to the standard curve of Trolox and expressed as μmol Trolox equivalents (TE)/g sample.

### 3.6. Cell Culture

Human hepatoma HepG2 cells were maintained in WME (Williams medium E) containing 5% FBS (fetal bovine serum), 10 mM Hepes, 2 mM L-glutamine, 5 μg/mL insulin, 0.05 μg/mL hydrocortisone and 1% antibiotic antimycotic solution. Human malignant melanoma A375 cells and human cervical carcinoma KB cells were maintained in DMEM (Dulbecco’s Modified Eagle’s Medium) containing 10% FBS and 1% antibiotic antimycotic solution.

### 3.7. Cellular Antioxidant Activity (CAA)

The CAA assay was followed as described previously with HepG2 cells [25,36]. HepG2 cells were cultured at 6 × 10^4^/well on a black-walled 96-well microplate. After incubating for 24 h at 37 °C, the growth medium was removed, and the cells were washed with PBS (pH 7.4). Then the cells were treated with 100 μL of appropriate concentrations of extract or quercetin standard dissolved in treatment medium containing 50 μM dichlorofluorescin diacetate (DCFH-DA) and maintained for 1 h. Subsequently, the medium was discarded and cells were treated with PBS wash or no PBS wash protocol. Finally, 600 μM ABAP in 100 μL of Hanks’ Balanced Salt Solution (HBSS) was added to produce fluorescent dichlorofluorescein (DCF). The fluorescence intensity was measured at 485 nm excitation and 535 nm emission by the FilterMax F5 Multi-Mode Microplate Reader every 5 min for 12 cycles at 37 °C. The CAA unit was calculated from the integrated area under the DCF fluorescence versus time curve, and the EC_50_ values were calculated from the median effect plot of log (CAA unit/(1-CAA unit)) versus log (dose). The CAA values were calculated by dividing the half maximal effective concentration (EC_50_) of quercetin standard by the EC_50_ of the extracts, which were expressed as μmol quercetin equivalents (QE)/100 g sample.

### 3.8. Antiproliferative Activity

The antiproliferative activity was evaluated as reported previously [37,38]. In brief, the cells in the growth media were seeded at a density of 2.5 × 10^4^/well on a 96-well microplate and incubated for 6 h at 37 °C. Then the medium was replaced by the growth medium containing various concentrations of phenolics extracts and incubated for 72 h at 37 °C. The number of viable cells after treatment was counted by methylene blue assay [38].

### 3.9. Statistical Analysis

The data were expressed as the mean ± standard deviation (SD) of at least triplicate measurements. The significant differences were analyzed using one-way ANOVA with Ducan`s assay. The *p* value less than 0.05 was considered statistically significant and marked with different letters. Correlation coefficients were calculated using Pearson`s correlation and SPSS 22.0 (SPSS Inc., Chicago, IL, USA). A dose-effect analysis was performed using Calcusyn software version 2.0 (Biosoft, Cambridge, UK).

## 4. Conclusions

In summary, results here indicated that there are many valuable chemical compounds distributed throughout the various parts of the *Boehmeria nivea* L. plant. Therefore, *Boehmeria nivea* L. is a good source of natural antioxidants and antiproliferative compounds with further application in food and pharmaceutical industries. Among the six vegetative parts compared, remarkable antioxidant and antiproliferative activities were found in xylem and bud. Besides, phloem and root also exhibited potent antioxidant capacity and notable antiproliferative activities, respectively. These results were consistent with and might be explained by various phenolic compositions such as chlorogenic acid, *p*-coumaric acid and epicatechin that can make these vegetative parts potential sources in industrial application, such as preservatives and food additives. Furthermore, bound phenolics should not be ignored and can contribute to the comprehensive utilization of *Boehmeria nivea* L.

## Figures and Tables

**Table 1:** Phenolic composition of different vegetative parts from *Boehmeria nivea* L.

Vegetative Parts		Phenolic Acids						Flavonoids			
		Chlorogenic Acid	Caffeic Acid	*P*-Coumaric Acid	Ferulic Acid	Gallic Acid	Benzoic Acid	Epicatechin	Rutin	Isoquercetin	Hyperoside
Root	Bound	14.10 ± 2.16 ^a^^*^	102.7 ± 1.8 ^h^	2087 ± 58 ^j^	31.30 ± 2.47 ^c^	10.15 ± 2.53 ^c^	nd ^#^	nd	12.93 ± 2.35 ^bc^	31.61 ± 2.86 ^b^	nd
	Free	43.48 ± 3.03 ^bc^	nd	60.86 ± 4.37 ^ab^	5.50 ± 0.18 ^a^	12.50 ± 1.38 ^c^	7.34 ± 1.16 ^a^	nd	35.07 ± 2.81 ^d^	nd	48.32 ± 2.75 ^d^
	Total ^†^	57.58 ± 1.78 ^c^	102.7 ± 1.8 ^h^	2148 ± 56 ^k^	36.80 ± 2.35 ^d^	22.64 ± 3.79 ^d^	7.34 ± 1.16 ^a^	nd	48.00 ± 5.16 ^e^	31.61 ± 2.86 ^b^	48.32 ± 2.75 ^d^
Xylem	Bound	nd	9.23±0.67 ^cd^	4114 ± 50 ^l^	74.19 ± 0.98 ^e^	nd	52.25 ± 1.38 ^c^	nd	29.60 ± 0.53 ^d^	42.59 ± 4.71 ^c^	16.31 ± 1.03 ^b^
	Free	1799 ± 25 ^h^	nd	41.19 ± 2.33 ^a^	5.11 ± 0.16 ^a^	1.92 ± 0.39 ^a^	41.87 ± 1.86 ^b^	2540 ± 61 ^c^	nd	nd	13.77 ± 0.69 ^b^
	Total	1799 ± 25 ^h^	9.23±0.67 ^cd^	4155 ± 52 ^l^	79.31 ± 1.06 ^f^	1.92 ± 0.39 ^a^	94.12 ± 3.16 ^e^	2540 ± 61 ^c^	29.60 ± 0.53 ^d^	42.59 ± 4.71 ^c^	30.09 ± 0.50 ^c^
Phloem	Bound	9.40 ± 0.60 ^a^	196.5 ± 2.4 ^k^	112.63 ± 1.58 ^c^	77.35 ± 1.17 ^ef^	nd	nd	32.49 ± 5.30 ^a^	30.67 ± 1.31 ^d^	nd	nd
	Free	743.3 ± 24.2 ^f^	2.70 ± 0.09 ^a^	53.78 ± 3.12 ^ab^	6.84 ± 0.06 ^a^	5.41 ± 0.56 ^b^	nd	1426 ± 74 ^b^	nd	nd	nd
	Total	752.7 ± 24.8 ^f^	199.2 ± 2.5 ^j^	166.4 ± 2.1 ^d^	84.18 ± 1.12 ^g^	5.41 ± 0.56 ^b^	nd	1459 ± 79 ^b^	30.67 ± 1.31 ^d^	nd	nd
Petiole	Bound	20.76 ± 1.24 ^ab^	4.87 ± 0.07 ^ab^	275.7 ± 3.3 ^e^	120.1 ± 3.8 ^j^	nd	nd	nd	10.31 ± 0.9 ^ab^	5.37 ± 0.10 ^a^	nd
	Free	66.59 ± 1.86 ^cd^	7.11 ± 0.34 ^bc^	68.38 ± 0.44 ^abc^	8.98 ± 0.15 ^a^	nd	nd	nd	8.51 ± 0.39 ^ab^	5.16 ± 0.03 ^a^	nd
	Total	87.35 ± 2.17 ^d^	11.98 ± 0.42 ^d^	344.1 ± 3.7 ^f^	129.1 ± 3.7 ^k^	nd	nd	nd	18.82 ± 0.64 ^c^	10.53 ± 0.12 ^a^	nd
Leaf	Bound	nd	8.36 ± 0.09 ^c^	367.74 ± 2.28 ^f^	90.41 ± 4.35 ^h^	nd	nd	nd	2.81 ± 0.13 ^a^	3.61 ± 0.03 ^a^	nd
	Free	121.0 ± 2.6 ^e^	7.83 ± 0.71 ^c^	62.14 ± 0.59 ^ab^	7.73 ± 0.14 ^a^	nd	nd	2.03 ± 0.37 ^a^	254.6 ± 9.57 ^h^	97.39 ± 4.16 ^d^	2.04 ± 0.18 ^a^
	Total	121.0 ± 2.6 ^e^	16.19 ± 0.80 ^e^	429.9 ± 1.8 ^g^	98.13 ± 4.33 ^i^	nd	nd	2.03 ± 0.37 ^a^	257.4 ± 9.69 ^h^	101.0 ± 4.2 ^d^	2.04 ± 0.18 ^a^
Bud	Bound	5.97 ± 0.11 ^a^	67.52 ± 1.44 ^f^	901.5 ± 10.4 ^h^	86.28 ± 4.5 ^gh^	2.68 ± 0.6 ^ab^	nd	3.47 ± 0.33 ^a^	9.23 ± 0.35 ^ab^	2.71 ± 0.03 ^a^	nd
	Free	1586 ± 29 ^g^	80.29 ± 3.00 ^g^	101.2 ± 0.6 ^bc^	13.46 ± 0.17 ^b^	nd	82.23 ± 0.96 ^d^	nd	135.1 ± 8.0 ^f^	160.5 ± 12.7 ^e^	nd
	Total	1592 ± 30 ^g^	147.8 ± 2.9 ^i^	1003 ± 11 ^i^	99.75 ± 4.33 ^i^	2.68 ± 0.6 ^ab^	82.23 ± 0.96 ^d^	3.47 ± 0.33 ^a^	144.3 ± 8.4 ^g^	163.2 ± 12.7 ^e^	nd

* Values (μg/g DW, n = 3) in the same column with different letters indicate significant differences among the samples (*p* < 0.05). ^#^ nd means not detected. ^†^ The total is the sum of free and bound content.

**Table 2:** The half maximal effective concentration (EC_50_ value) of soluble free fractions of different vegetative parts from *Boehmeria nivea* L.

	A375	KB	HepG2
Root	3.11 ± 0.10 ^b^^*^	1.30 ± 0.07 ^b^	1.41 ± 0.07 ^a^
Xylem	2.84 ± 0.08 ^b^	1.64 ± 0.01 ^c^	0.96 ± 0.02 ^a^
Phloem	11.06 ± 0.43 ^c^	3.62 ± 0.22 ^d^	1.09 ± 0.07 ^a^
Petiole	ND ^#^	ND ^#^	2.28 ± 0.35 ^b^
Leaf	ND ^#^	ND ^#^	3.46 ± 0.54 ^c^
Bud	0.06 ± 0.01 ^a^	0.13 ± 0.01 ^a^	0.92 ± 0.14 ^a^

* Values (mg dry weight, DW/mL) in the same column with different letters indicate significant differences among the samples (*p* < 0.05). ^#^ ND means not detected at the doses of soluble free extracts (petiole: 8 mg DW/mL; leaf: 10 mg DW/mL).
